# An odorant receptor from *Anopheles sinensis* in China is sensitive to oviposition attractants

**DOI:** 10.1186/s12936-018-2501-4

**Published:** 2018-10-05

**Authors:** Hongmei Liu, Luhong Liu, Peng Cheng, Xiaodan Huang, Maoqing Gong

**Affiliations:** 1 Department of Medical Entomology, Shandong Institute of Parasitic Diseases, Shandong Academy of Medical Sciences, Jining, 272033 Shandong People’s Republic of China; 2 Jining Center for Disease Control and Prevention, Jining, 272033 Shandong People’s Republic of China

**Keywords:** *Anopheles sinensis*, AsinOrco, AsinOR10, 3-Methylindole

## Abstract

**Background:**

*Anopheles sinensis* is an important vector for the spread of malaria in China. Olfactory-related behaviours, particularly oviposition site seeking, offer opportunities for disrupting the disease-transmission process.

**Results:**

This is the first report of the identification and characterization of AsinOrco and AsinOR10 in *An. sinensis*. AsinOrco and AsinOR10 share 97.49% and 90.37% amino acid sequence identity, respectively, with related sequences in *Anopheles gambiae*. A functional analysis demonstrated that AsinOrco- and AsinOR10-coexpressing HEK293 cells were highly sensitive to 3-methylindole, but showed no significant differences in response to other test odorants when compared to DMSO.

**Conclusions:**

AsinOrco was characterized as a new member of the Orco ortholog subfamily. AsinOR10, which appears to be a member of the OR2-10 subfamily, is directly involved in identification of oviposition sites. This finding will help to elucidate the molecular mechanisms underlying olfactory signaling in *An. sinensis* and provide many more molecular targets for eco-friendly pest control.

**Electronic supplementary material:**

The online version of this article (10.1186/s12936-018-2501-4) contains supplementary material, which is available to authorized users.

## Background

Malaria is one of the most important infectious diseases seriously endangering human health and safety. The World Health Organization (WHO) lists malaria with AIDS and tuberculosis as the top three public health problems globally. Malaria is also one of the most important mosquito-borne diseases in China. To respond proactively to the global action to eliminate malaria, China launched the Malaria Action Plan [1] in 2010, which clearly states that “by 2015, the country except for some border areas of Yunnan and other areas have no local malaria cases”; “by 2020, the national malaria elimination.” Currently, most counties (districts) in China have completed an assessment of malaria elimination. However, conditions are still favourable for the spread of malaria in some regions; even if the source of infection can be discovered and cleared in a timely, there is still a risk of local transmission and epidemic rebound. With rapid globalization and implementation of the national “Belt and Road” initiative, the number of people visiting areas of high malaria transmission, such as Africa and Southeast Asia, for business, employment and tourism purposes has increased significantly. As a result, the proportion of overseas imported cases, which reached 99.9% (3317/3321) in 2016, shows an increasing trend [2]. Such an increase poses a potential risk to relatively stable malaria-endemic areas. For example, a short-term and large-scale clustered imported outbreak occurred in Guangxi Province in 2013 [3]. In addition, malaria-nonendemic areas lack diagnostic awareness of imported malaria cases, and severe illness and death can occur.

*Anopheles sinensis*, with a wide distribution and a large population, is an important vector for the spread of malaria in China. The main strategy for the elimination of malaria by the WHO is the timely and effective removal of infection sources and preventing spread among epidemic sites. Given the resistance of *An. sinensis* populations to commonly used insecticides, alternative control methods are crucially needed. Researchers have combined *Bacillus thuringiensis* var. *israelensis* with oviposition attractants in “attract-and-kill” strategies [4] to collect more gravid females [5] and [6] eggs than with control traps. As mosquitoes use their olfactory system to search for oviposition sites, research on these systems is of key importance.

The olfactory system of insects mainly includes olfactory receptors (ORs), odorant-binding proteins (OBPs) and olfactory receptor neurons (ORNs). Previous studies have demonstrated that ORs can convert odour-stimulating chemical signals into electrical signals and transmit nerve impulses to the dendrites of olfactory neurons [7]. Accordingly, ORs are involved in mating, blood sucking, oviposition site searching and other important life activities of mosquitoes.

ORs in insect olfactory sensory neurons (OSNs) include a coreceptor designated Orco (OR7) and conventional ligand-binding odorant receptors (ORXs). Orco genes from different species are highly conserved [8, 9]. Other highly divergent ORs are conventional odorant receptors, correlating with some olfactory-mediated behavioural functions [10], and these ORs have been associated with certain biological information about odorants [11]. Consistently, AgamOR2, AgamOR5, AgamOR8 and AgamOR65 [12] are narrowly tuned to indole, 2,3-butanedione, 1-octen-3-ol, and 2-ethylphenol, respectively. In addition, some ORs respond strongly to specific odorants; for example, CquiOR10 [13] has been shown to respond strongly to 3-methylindole [14], an oviposition site volatile attractant, whereas AgamOR10 [12, 15] is highly sensitive to 3-methylindole and indole. Indole [12, 16] is a volatile attractant component of both human sweat and oviposition sites. In the previous research, AablOR10 was linked to host- and oviposition-seeking behaviours, prompting us to examine the odorant response profile of AsinOR10. This study identified AsinOrco and AsinOR10 of *An. sinensis* and examined the odorant response profile of AsinOR10.

## Methods

### Mosquito rearing and blood feeding

*Anopheles sinensis* (laboratory-susceptible strain) larvae and pupae were reared on yeast powder, and adults were maintained on a 10% sugar solution at 25–27 °C and 70–80% relative humidity with a photoperiod of 12:12 h. Three-day-old adult females were blood-fed on a human volunteer arm using standard protocols [17].

### Identification of putative AsinOrco sequences

Predicted amino acid sequences of *An. sinensis* (ASIS023681-RA) [18], *Anopheles funestus* (KF819859), *Anopheles gambiae* (AGAP002560-RA) and *Culex pipiens quinquefasciatus* (DQ231246) Orco orthologs were obtained from VectorBase. Primers (Additional file 1: Table S1) used for two-step RT-PCR were first designed based on these sequences using primer 5.0 to amplify partial gene sequences of AsinOrco and AsinOR10. Total RNA extraction from female adult mosquitoes (3–7 days old) and cDNA synthesis were performed using an RNeasy Mini Kit (QIAGEN, Hilden, Germany), the TURBO DNA-free™ Kit (Ambion, Carlsbad, CA, USA) and TaKaRa PrimeScript™ RT-PCR Kit (Takara, Otsu, Shiga, Japan) following the manufacturers’ instructions. Gene-specific primers (Additional file 1: Table S1) were then designed for 5′- or 3′-end rapid amplification of cDNA ends (RACE) to amplify full-length coding sequences using a SMARTer™ RACE cDNA amplification kit (Clontech, Mountain View, CA, USA).

### Identification of putative AsinOR10 sequences

Nested RT-PCR primers (Additional file 1: Table S1) were designed based on the predicted amino acid sequences of *An. sinensis* (ASIC007209-RA) [18], *Culex pipiens* (FJ008065), *Culex quinquefasciatus* (GU945397), *Anopheles quadriannulatus* (FJ008069), *An. gambiae* (AGAP009520-RA) and *Anopheles stephensi* (FJ008074) OR10 orthologs. PCR was carried out using TaKaRa Tks Gflex DNA Polymerase (Takara, Otsu, Shiga, Japan). PCR amplification products were examined by 1.5% agarose gel electrophoresis and verified by DNA sequencing (Invitrogen, Shanghai, China). The obtained sequences were compared with predicted AsinORs and AgamORs using DNAMAN.

### Sequence analysis

Amino acid sequences of ORs were aligned using the program ClustalW, and the neighbor-joining tree was built using the MEGA 5.0 program [19]. The membrane topology of the OR sequences was predicted using the HMMTOP (version 2.0) and TMHMM (version 2.0) [20] servers.

### Expression of AsinORs in HEK293 cells

The full-length coding sequences (CDSs) of AsinORs were cloned into the pME18s mammalian expression plasmid [9] using specific primers (Additional file 1: Table S1). The DsRed coding sequence was amplified from pIRES2-DsRed plasmids (Clontech, Mountain View, CA, USA) using primers containing the appropriate restriction sites. AsinORs were cloned into the pME18s plasmid in-frame with the DsRed coding sequence [8]. HEK293 (human embryo kidney 293) cells (purchased from the Chinese Academy of Sciences) were cultured in an incubator at a constant temperature of 37 °C with 5% CO_2_ and transiently transfected with AsinORs using Lipofectamine^®^ 2000 Reagent (Invitrogen, Carlsbad, CA) [21]. Expression of ORs was confirmed by RT-PCR after 24 h; subcellular location analysis and western blotting were performed after 48 h. The two-step RT-PCR primers and nested RT-PCR primers are provided in Additional file 1: Table S1. Cells were lysed with RIPA buffer (50 mM Tris, pH 7.5, 150 mM NaCl, 1 mM EDTA, 0.25% sodium deoxycholate, 0.1% Triton X-100, 1% Nonidet P-40). The lysates were mixed with in SDS–PAGE buffer (62.5 mM Tris, pH 6.8, 2% SDS, 5% 2-mercaptoethanol, 10% glycerol, 0.02% bromophenol blue), heated at 95 °C for 5 min, separated by 10% SDS-PAGE gel electrophoresis and transferred to a PVDF membrane (Immobilon^TM^-P, Millipore). The blot was washed with TBST, incubated with 5% skim milk for 60 min, and incubated overnight with an anti-RFP antibody (Abcam, Cambridge, US) raised in mice at a dilution ratio of 1:1000 in 1 × PBS or anti-GAPDH antibody (Abcam, Cambridge, US) at 1:3000 dilution at 4 °C. The blot was then incubated with a horseradish peroxidase (HRP)-conjugated anti-mouse IgG secondary antibody (1:4000) (Bethyl Laboratories, Montgomery, TX, USA) at room temperature for 90 min.

### Calcium-imaging assay

Forty-eight hours after transfection, AsinOR-expressing cells were rinsed three times with HBSS. Fluo4-AM (Dojindo Laboratories, Tokyo, Japan) at a concentration of 2 μM was added, and the cells were incubated for 30 min at 37 °C in the dark. The cells were rinsed three times with HBSS before the addition of fresh HBSS (containing Ca^2+^) [8] and tested using a panel of odorants, including indole, 1-octen-3-ol, 1-methylindole, 3-methylindole, 2-methylphenol, 2,3-butanedione, 2-ethylphenol, and dimethyl sulfoxide (Sigma). All odorants (≥ 98% pure) were dissolved in DMSO and added to a final concentration of 10^−6^ M.

Fluorescence images were acquired using a laser scanning confocal microscope (Olympus, Japan). The Ca^2+^ level is represented as relative fluorescence change (ΔF/F_0_), where ΔF is the difference in peak fluorescence caused by stimulation and F_0_ is the baseline fluorescence [22, 23]. Baseline fluorescence was measured 100 s prior to adding the chemicals. Responses were quantified by the mean values of the maximal elevations (ΔF/F_0_) [8]. Each odorant was assayed in triplicate per dish, and at least seven cells per dish were selected randomly. All assays were performed in triplicate.

### Statistical analysis

Statistical analyses of differences in the cellular experimental results were conducted with one-way ANOVA followed by post hoc Tukey HSD tests (homogeneity of variance: *P *> 0.05).

## Results

### Identification of putative AsinOR genes

**Fig. 1:**
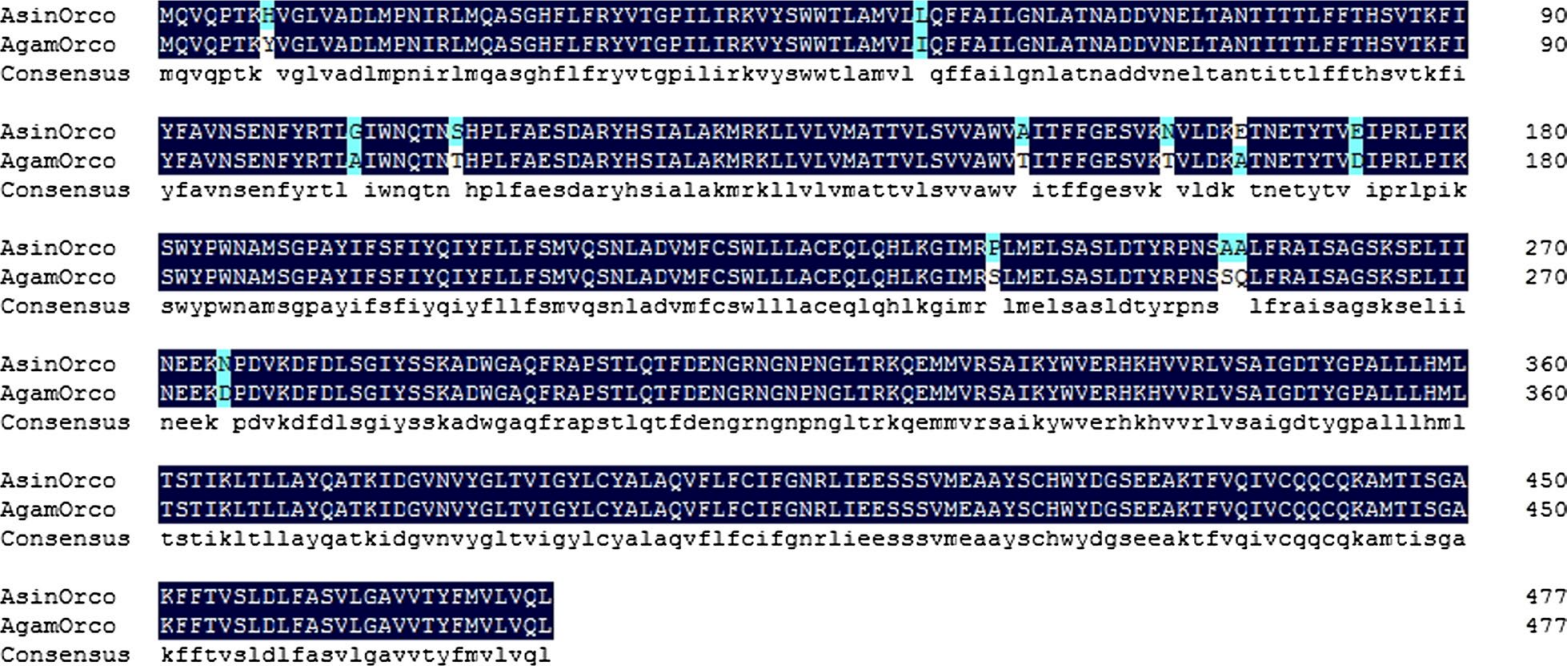
Alignment of mosquito Orco amino acid sequences. Dark blue shading indicates residues conserved between AsinOrco and AgamOrco

**Fig. 2:**
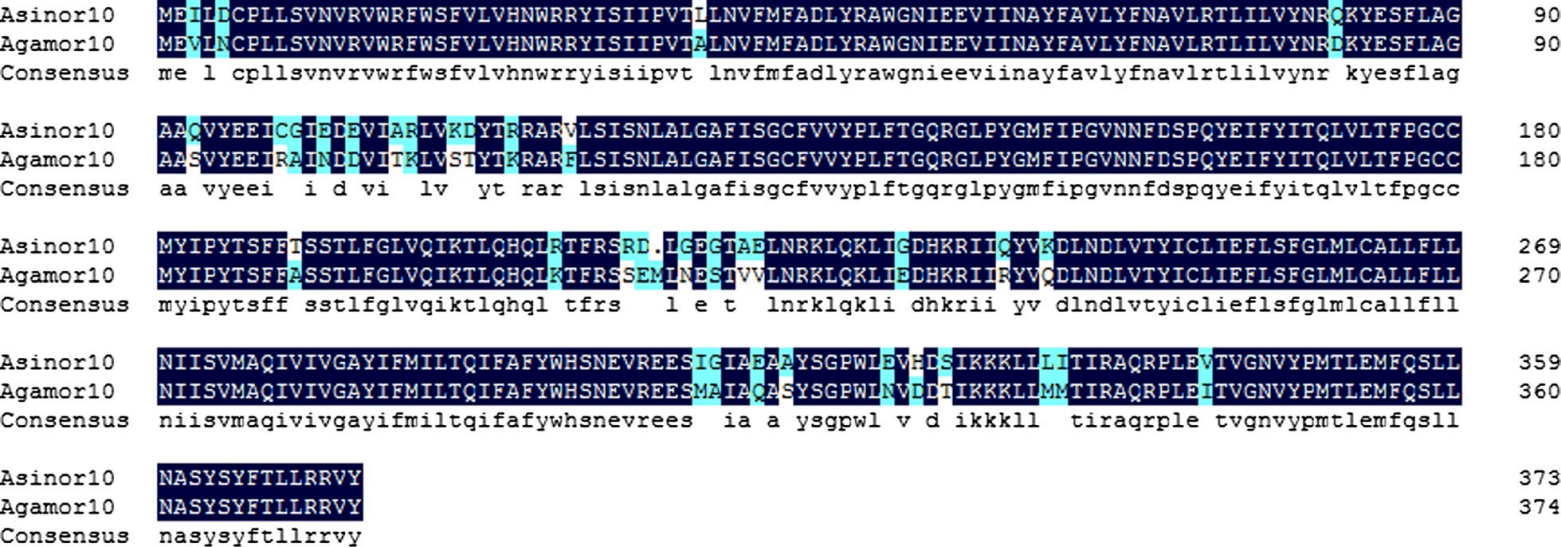
Alignment of mosquito OR10 amino acid sequences. Dark blue shading indicates residues conserved between AsinOR10 and AgamOR10

Full-length coding sequences for AsinOrco and AsinOR10 were successfully obtained based on bioinformatics and homologous genes. AsinOrco, which is 1437 bp in length and encodes 479 amino acids, exhibits 96.66% sequence identity with predicted AsinOrco (98.96%) and AgamOrco (90.61%). Similarly, AsinOR10, which is 1125 bp in length and encodes 375 (93.35%) amino acids, shares 100% and 80.59% identity with predicted AsinOR10 and AgamOR10, respectively. An alignment of AsinOrco (97.49%) and AsinOR10 (90.37%) amino acid sequences with related sequences in *An. gambiae* is shown in Figs. 1, 2. In general, ORs display a high level of divergence [24]. An interesting phenomenon is that the ORs from different species have very high sequence conservation.

### Sequence analysis

**Fig. 3:**
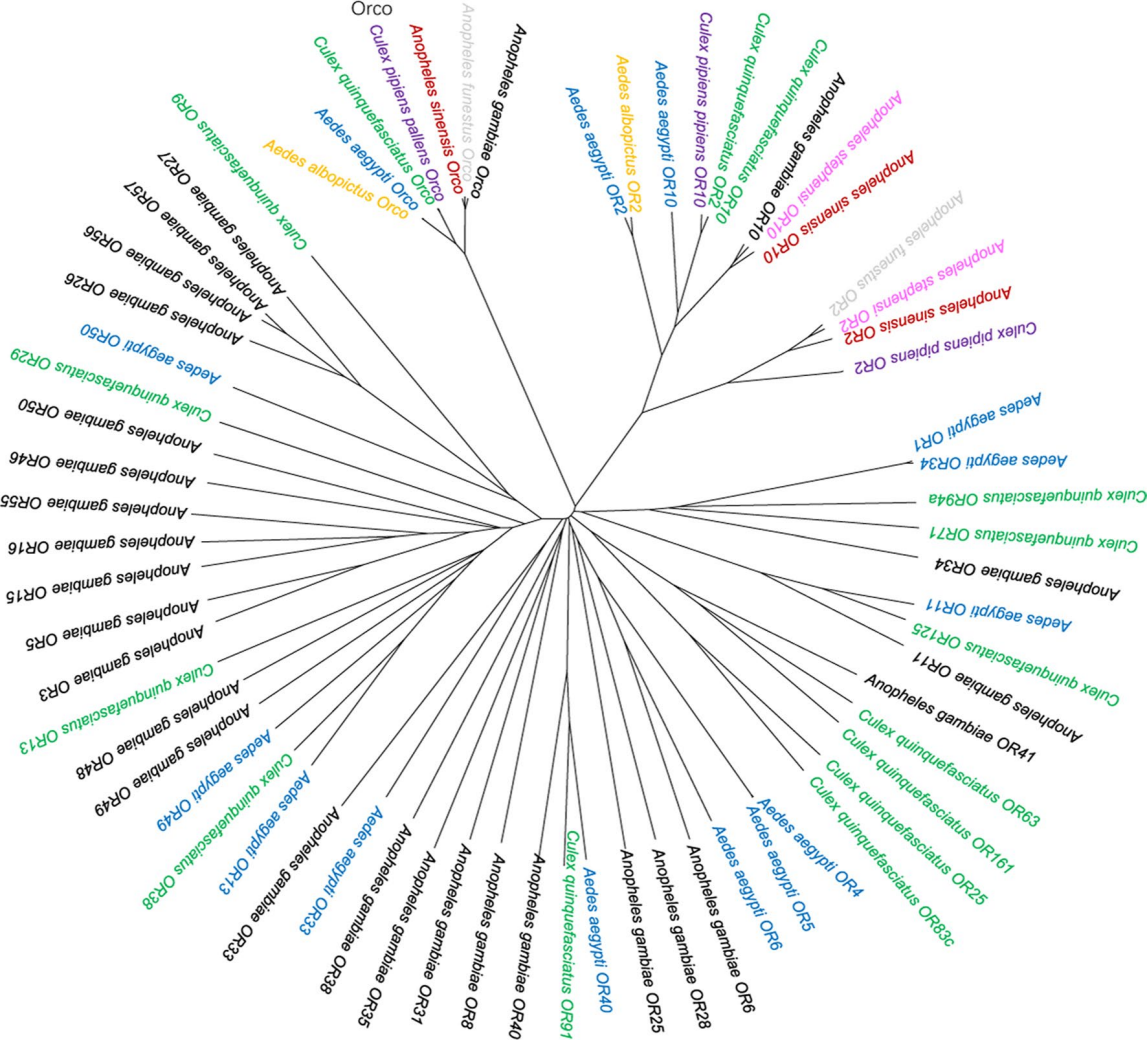
Phylogenetic relationships of mosquito ORs. *Anopheles sinensis* ORs are in red, *Anopheles gambiae* ORs are in black, *Anopheles stephensi* ORs are in pink, *Anopheles funestus* ORs are in light gray, *Culex quinquefasciatus* ORs are in green, *Culex pipiens pipiens* ORs are in purple, *Aedes aegypti* ORs are in blue, and *Aedes albopictus* ORs are in yellow. AsinOrco was grouped into the coreceptor subfamily, and AsinOR10 was grouped into the OR2-10 subgroup

To explore relationships among ORs from different species, phylogenetic tree analysis was carried out using similar OR sequences, mainly including AgamORs, AaegORs and CquiORs. The results revealed the existence of different subgroups (Fig. 3). For this study, AsinOrco and AgamOrco, AfunOrco, AalbOrco, AaegOrco, CquiOrco and CppOrco were found to be clustered together. This finding indicates that AsinOrco belongs to the coreceptor subfamily, whereas AsinOR10, which is identified as a conventional odorant receptor, clusters with the OR2-10 subgroup. Among them, AsinOrco and AsinOR10 display the highest identity with AgamOrco/AfunOrco and AgamOR10/AsteOR10, respectively. Significantly, with the exception of OR7 orthologs, OR2 and OR10 are the most conserved ORs in the phylogenetic tree. This sequence conservation suggests that OR10 may show an odorant-induced response profile similar to that of OR2. These interesting phenomena encouraged us to examine the odorant response profile of AsinOR10.

**Fig. 4:**
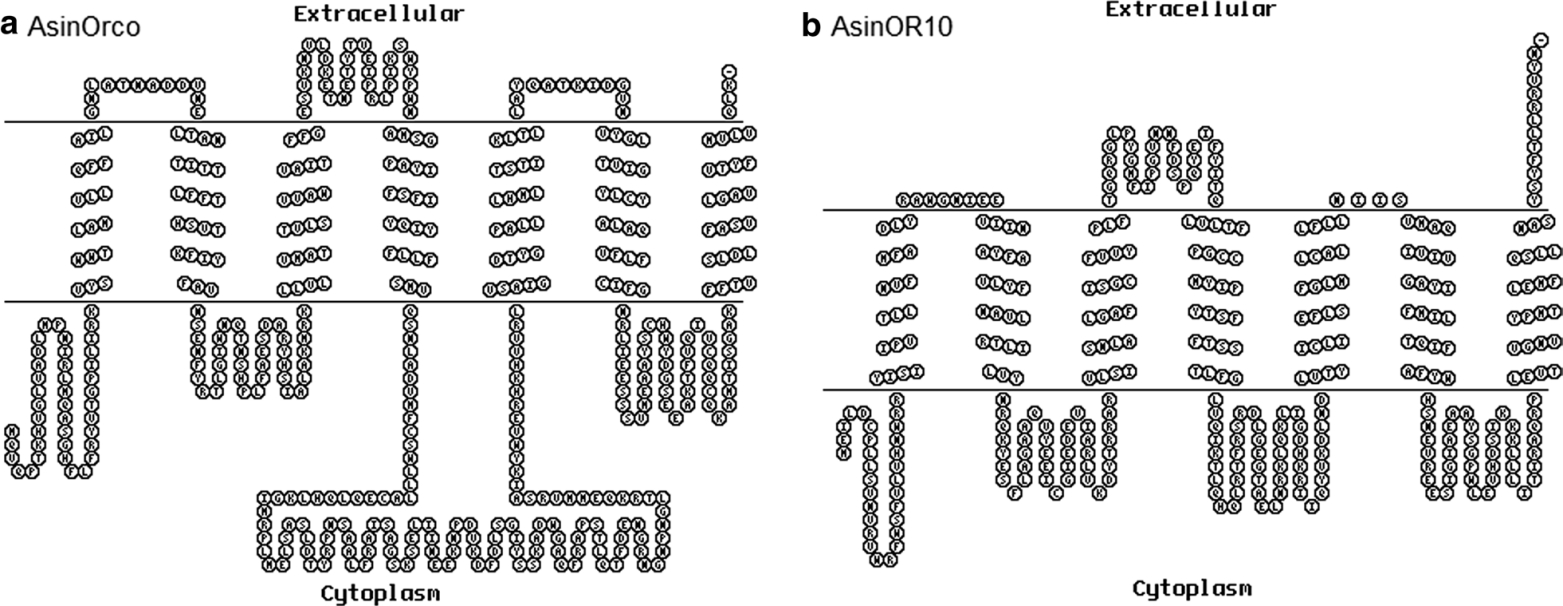
Transmembrane regions of AsinORs predicted using HMMTOP and TMHMM. **a** Transmembrane regions of AsinOrco. **b** Transmembrane regions of AsinOR10

Membrane topology predictions for AsinOrco and AsinOR10 revealed that these receptors belong to the seven-transmembrane (TM) protein family with an intracellular amino-terminus (Fig. 4). Analysis of the primary amino acid sequence of AsinOrco shows that it contains a putative calmodulin (CaM)-binding site (^328^SAIKYWVER^336^) identified in DmelOrco (^336^SAIKYWVER^344^) and in AalbOrco (^329^SAIKYWVER^337^) [8]; in contrast, AsinOR10 does not have this putative CaM-binding site or channel gate sequences. The observed sequence conservation supports our hypothesis that AsinOrco may form a channel gate, as reported for DmelOrco [25, 26], and that it may form complexes involved in odour signal transduction [20, 25].

### Heterologous expression of AsinOR10 in HEK293 cells

**Fig. 5:**
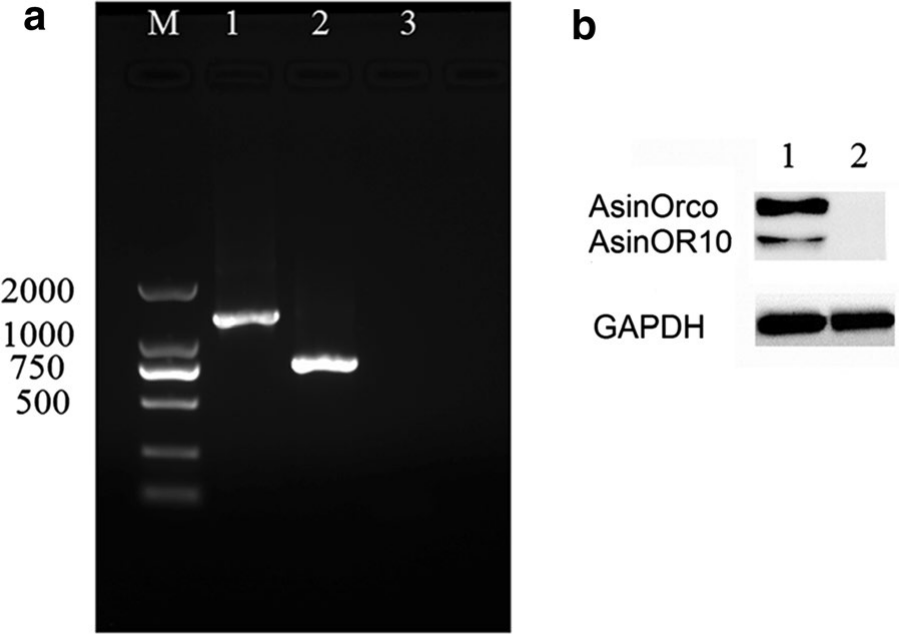
RT-PCR and western blotting. **a** AsinORs transcripts detected by RT-PCR in HEK293 cells at 24 h after transfection. Lane M: molecular weight marker in the 2000 bp series, Lane 1: AsinOrco, Lane 2: AsinOR10, Lane 3: pME18s plasmid. **b** AsinORs proteins detected by western blotting of HEK293 cells at 48 h after transfection. Lane 1 recombinant AsinOrco-DsRed and AsinOR10-DsRed detected with anti–RFP antibody, Lane 2: pME18s plasmid

**Fig. 6:**
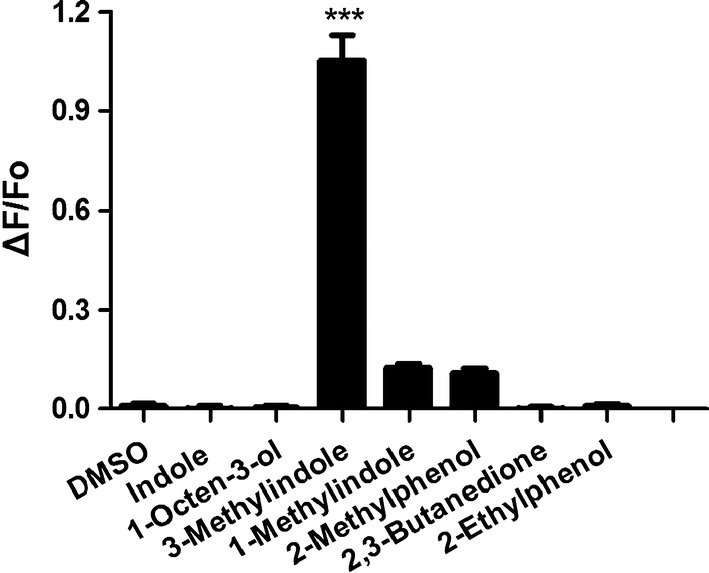
HEK293 cells coexpressing AsinOrco and AsinOR10 were challenged with a panel of odorant compounds. All odorants were added to a final concentration of 10^−6^ M. The Ca^2+^ level is represented as the relative fluorescence change (ΔF/F_0_), where ΔF is the difference in peak fluorescence caused by stimulation and F0 is the baseline fluorescence. Responses were quantified by the mean values of the maximal elevations (ΔF/F_0_). Maximal intracellular calcium concentrations occurred in HEK293 cells coexpressing AsinOrco and AsinOR10 (*F*_(7, 461)_ = 120.240, *P *< 0.005; Dunnett T3 vs DMSO, 3-methylindole: *P *< 0.005; indole: *P *= 1.000; 1-octen-3-ol: *P *= 1.000; 1-methylindole: *P *= 0.188; 2-methylphenol: *P *= 0.320; 2,3-butanedione: *P *= 1.000; 2-ethylphenol: *P *= 1.000)

AsinOR transcripts were detected in HEK293 cells at 24 h (Fig. 5a), and corresponding proteins at approximately 78 kDa and 67 kDa (Fig. 5b) were identified by western blotting at 48 h. The previous study [8] found that individual OR proteins respond weakly to certain test chemicals but that HEK293 cells coexpressing Orco and OR respond strongly. Therefore, HEK293 cells coexpressing AsinOrco and AsinOR10 were screened using a panel of odorants at a final concentration of 10^−6^ M (Fig. 6) in calcium-imaging experiments. The strongest fluorescence was elicited by 3-methylindole (skatole) (measured as the relative fluorescence change, ΔF/F0). Interestingly, except for 3-methylindole (*F*_(7, 461)_ = 120.240, *P *< 0.005; Dunnett T3 vs DMSO, 3-methylindole: *P *< 0.005; indole: *P *= 1.000; 1-octen-3-ol: *P *= 1.000; 1-methylindole: *P *= 0.188; 2-methylphenol: *P *= 0.320; 2,3-butanedione: *P *= 1.000; 2-ethylphenol: *P *= 1.000), the receptors showed no significant differences in their responses to other odorants compared to dimethyl sulfoxide (DMSO). The study interprets these results to indicate that AsinOR10 has high sensitivity for 3-methylindole but very low sensitivity for indole and other methylindoles, including 1-methylindole.

## Discussion

This study is the first report of the identification and characterization of AsinOrco and AsinOR10. Although ORs typically display a high level of divergence [24], AsinOrco and AsinOR10 share 97.49% and 90.37% amino acid sequence identity with the coreceptor and OR2-10 subfamilies, respectively. This study utilized the nomenclature for Orco [27] and found that AsinOrco exhibits at least 50% sequence identity with orthologs from other insect species, and the predicted protein size is larger than that of conventional ORs. Membrane topology predictions show that AsinOrco and AsinOR10 belong to the TM7 protein family and have an intracellular amino-terminus. In addition, AsinOrco has the putative CaM-binding site (^328^SAIKYWVER^336^) identified in DmelOrco (^336^SAIKYWVER^344^) and in AalbOrco (^329^SAIKYWVER^337^) [8]. This conservation of structure may also account for functional similarity. Overall, identification and functional validation of Orco orthologs are hot research topics. In the previous study, AalbOrco was demonstrated to transmit olfactory signaling, but did not recognize odorants [8]. In fact, Orco forms a complex with conventional odorant receptors and is essential for odour signal transduction [20]. Indeed, silencing or mutation of Orco [8, 28, 29] damages normal odorant responses. Notably, the function of Orco is so similar that some researchers [21] have even used *Drosophila melanogaster* Orco as a heterodimerization partner to examine the function of AalbORs. In this study, AsinOrco was characterized as a new member of the Orco ortholog subfamily. Furthermore, HEK293 cells coexpressing AsinOrco and AsinOR10 responded to odorants.

Conventional OR sequence homology has often been associated with odorant specificity [21, 30, 31], and the narrow OR response to odorants may be highly relevant to mosquito ecology [12]. In previous studies, OR2-10 orthologs [12, 13, 21, 30] were found to be more likely to be highly sensitive to indole and 3-methylindole, attractants of oviposition sites, therefore, this study focused on the ability of AsinOR10 to perceive oviposition attractants. AsinOrco- and AsinOR10-coexpressing cells were exposed to seven odorants, including indole, 1-methylindole, 3-methylindole, 1-octen-3-ol, 2-methylphenol, 2,3-butanedione, and 2-ethylphenol. Indole [12, 16] is a volatile attractant of oviposition sites and human sweat. 3-Methylindole [14, 32, 33], also known as skatole, is a ubiquitous oviposition site volatile attractant and an egg raft pheromone; 1-methylindole is another methylindole compound. 1-Octen-3-ol [33], a volatile attractant from large herbivores and humans, is known to attract some anophelines [33, 34], and 2-methylphenol [30], identified as the best ligand among phenols, elicits a strong electrophysiological response from CquiOR2. 2,3-Butanedione [35] is a metabolic byproduct of human skin microflora, which excites narrowly tuned AgamOR5 [12], and 2-ethylphenol [36] is found in the urine of animals and evokes a strong electrophysiological response from AgamOR65 [12].

In contrast to DMSO, 3-methylindole elicits a fluorescence reaction (measured as relative fluorescence change, ΔF/F0). This finding is similar to previous results showing that CquiOR10 [13, 30], AalbOR10 [8] and AgamOR10 [12] orthologs respond sensitively to 3-methylindole and thus further confirm the functional conservation of OR10 orthologs. Regardless, CquiOR10 [13, 30], AalbOR10 [8] and AgamOR10 [12] responded to a set of aromatic compounds, including each of the methylindoles, 1-octen-3-ol and indole, using the *Xenopus* Oocyte System or the *Drosophila melanogaster* “empty neuron” system, whereas AsinOR10 showed no significant differences in responses to indole, 1-octen-3-ol and 1-methylindole compared to DMSO in HEK293 cells. These results might be due to differences in the intracellular epitope tags of these systems, which may influence the selectivity of the receptor, or this OR might not be responsive to the chemicals tested. Despite the use of a heterogeneous expression system, the results indicate that AsinOR10 is directly involved in oviposition site-seeking behaviour.

## Conclusions

In summary, AsinOrco was characterized as a new member of the Orco ortholog subfamily, and AsinOR10 was found to be a member of the OR2-10 subfamily. AsinOR10 is directly involved in oviposition site identification. These results will help in exploration of the molecular mechanism underlying the olfactory signal transduction pathway in *An. sinensis* and provide more molecular targets for eco-friendly pest control.

## Additional file

**Additional file 1: Table S1.** List of oligonucleotide primers.
